# Whole-Genome Sequence Analysis of Postpandemic Influenza A(H1N1)pdm09 Virus Isolates from India

**DOI:** 10.1128/genomeA.00727-13

**Published:** 2013-09-19

**Authors:** Minal Dakhave, Atul Khirwale, Kunal Patil, Archana Kadam, Varsha Potdar

**Affiliations:** National Institute of Virology, Pune, Maharashtra, India

## Abstract

The pandemic influenza A(H1N1)pdm09 virus was first detected in India in May 2009 and continued to circulate in the postpandemic period. Whole-genome sequence analysis of postpandemic A(H1N1)pdm09 viruses showed the circulation of clade 6 and clade 7 viruses. The hemagglutinin (HA) gene showed increased diversity compared with that in the pandemic phase.

## GENOME ANNOUNCEMENT

After the first reported case of infection with the influenza A(H1N1)pdm09 virus in India (1), the virus was found to be in circulation in many parts of the country. A previous report (2) of 31 whole-genome sequences of the A(H1N1)pdm09 virus during a pandemic revealed higher substitution rates than those observed globally. In the postpandemic period, the A(H1N1)pdm09 virus remained in circulation and showed unusually increased activity and severity in March and April, especially in the northern and western parts of the country, for three consecutive years, which is unseasonal for India. The National Institute of Virology in Pune carried out diagnostic and genetic analyses as part of postpandemic surveillance (institutional review board [IRB] approved). Thirteen whole-genome sequences from isolates obtained from April 2011 to April 2013 were studied and compared with the 31 whole-genome sequences from the pandemic period (2) to identify the genomic changes responsible for pathogenesis and drug susceptibility of A(H1N1)pdm09.

During the study period, 6,836 samples were analyzed, and of the 1,675 real-time PCR positives, 1,036 (61.8%) were A(H1N1)pdm09, 242 (14%) were A(H3N2), and 397 (23%) were type B positive. Isolation was carried out with MDCK cells. Thirteen A(H1N1)pdm09 isolates from a period of unusual activity (2 isolates from 2011, 8 isolates from 2012, and 3 isolates from 2013) were selected for whole-genome sequencing. All the eight influenza gene segments were amplified in an overlapping manner by one-step conventional reverse transcriptase PCR (RT-PCR) using the whole-genome primers recommended by the WHO and the CDC (3). Sequencing was carried out by using the BigDye Terminator version 3.1 cycle sequencing ready reaction kit (ABI) and processing for capillary electrophoresis on an ABI 3730 DNA analyzer; sequences were curated using Sequencing Analysis version 5.3, and MEGA version 5.2 was used for sequence alignments.

All gene segments were compared with those of the vaccine component strain A/California/07/2009, along with those of the globally circulating strains. The result showed that A(H1N1)pdm09 viruses of clade 7 (D97N mutation) and clade 6 (A197T mutation) were in circulation. A single isolate from 2013 possessed D222N (D239N) in the receptor binding region of the hemagglutinin (HA) molecule (4). The HA gene mutations P83S, S84G, S183P, S185T, S203T, R223Q, E374K S451N, and I321V observed during the pandemic were also noted in the postpandemic period (1, 2). Newly identified mutations in HA were S143G, K283E, and E499K. Increased diversity in the HA gene was observed from 2011 to 2013. All isolates remained sensitive to neuraminidase inhibitor (NAI) drugs and showed N369K and V241I amino acid changes in the neuraminidase (NA) gene, which may facilitate the stability of resistant viruses (5). Similarly, mutations noticed for the polymerase basic 2 (PB2) gene were D195N, V731I, and N456S; for PB1, the mutations were G154D, I397M, and I435T; for the polymerase acidic (PA) gene, they were V100I and I330V; for M1, K239R; for M2, D21G; for the nonstructural 1 (NS1) gene, L90I, N205S, and I111M/T; and for NS2, T48A and V49M.

Continued molecular surveillance and whole-genome sequencing are important for understanding significant evolutionary changes in this pandemic virus.

### Nucleotide sequence accession numbers.

The whole-genome sequences of 13 Indian A(H1N1) pdm09 isolates from the period 2011 to 2013 have been deposited in GenBank under accession no. KF280652 to KF280755; accession numbers are listed in Table 1.

**Table 1:** Accession numbers of whole-genome sequences of A(H1N1) pdm09 isolates

	GenBank accession no. by gene^*a*^:							
Isolate	PB2	PB1	PA	HA	M	NP	NA	NS
NIV1112874	KF280652	KF280653	KF280654	KF280655	KF280656	KF280657	KF280658	KF280659
NIV1114854	KF280660	KF280661	KF280662	KF280663	KF280664	KF280665	KF280666	KF280667
NIV12388	KF280668	KF280669	KF280670	KF280671	KF280672	KF280673	KF280674	KF280675
NIV121716	KF280676	KF280677	KF280678	KF280679	KF280680	KF280681	KF280682	KF280683
NIV121717	KF280684	KF280685	KF280686	KF280687	KF280688	KF280689	KF280690	KF280691
NIV121773	KF280692	KF280693	KF280694	KF280695	KF280696	KF280697	KF280698	KF280699
NIV121778	KF280700	KF280701	KF280702	KF280703	KF280704	KF280705	KF280706	KF280707
NIV121939	KF280708	KF280709	KF280710	KF280711	KF280712	KF280713	KF280714	KF280715
NIV12946	KF280716	KF280717	KF280718	KF280719	KF280720	KF280721	KF280722	KF280723
NIV122268	KF280724	KF280725	KF280726	KF280727	KF280728	KF280729	KF280730	KF280731
NIV132467	KF280732	KF280733	KF280734	KF280735	KF280736	KF280737	KF280738	KF280739
NIV131845	KF280740	KF280741	KF280742	KF280743	KF280744	KF280745	KF280746	KF280747
NIV132194	KF280748	KF280749	KF280750	KF280751	KF280752	KF280753	KF280754	KF280755

a PB2, polymerase basic 2; PA, polymerase acidic; HA, hemagglutinin; M, matrix; NP, nucleoprotein; NA, neuraminidase; NS, nonstructural.
